# Optimizing Wound Care: The Mechanistic Role of Dressing Change Frequency in Acute and Diabetic Wound Healing

**DOI:** 10.1111/jocd.70643

**Published:** 2026-01-02

**Authors:** Ping He, Yiwen Han, Chencheng Mo, Hantao Li, Xiangdong Qi

**Affiliations:** ^1^ Department of Plastic & Aesthetic Surgery Center, Zhujiang Hospital Southern Medical University Guangzhou Guangdong Province People's Republic of China

**Keywords:** acute wound, diabetic wound, inflammatory microenvironment, macrophage polarization, precision wound management, therapeutic frequency, wound healing

## Abstract

**Background:**

Dressing change frequency is fundamental to wound care yet governed by tradition, creating a clinical paradox: protecting acute wounds versus debriding diabetic, biofilm‐laden ones. Because “one‐size‐fits‐all” protocols ignore this pathophysiological divide, the mechanistic principles for tailoring frequency to the specific wound state remain critically undefined.

**Methods:**

We established parallel full‐thickness wound models in normal (acute) and diabetic mice, randomized to high‐, intermediate‐, or low‐frequency dressing change regimens. Healing was evaluated via macroscopic closure, histology, and qRT‐PCR analysis of key genes regulating inflammation, macrophage polarization, and matrix remodeling.

**Results:**

An intermediate‐frequency regimen drove superior healing in acute wounds, with accelerated closure and organized collagen deposition. In stark contrast, diabetic wounds required high‐frequency treatment; low‐frequency intervention was actively detrimental. These opposing outcomes were governed by a common mechanism: the optimal frequency orchestrated a rapid resolution of LBP‐mediated inflammation and triggered a decisive M1‐to‐M2 macrophage shift, driving effective collagen synthesis.

**Conclusions:**

This study challenges the static paradigm in wound care. We establish that dressing frequency is a dynamic therapeutic variable that must be tailored to the wound's pathophysiology. Our findings provide the first evidence‐based rationale for modulating intervention rhythm based on a wound's inflammatory state—a critical strategy to accelerate acute repair and rescue diabetic nonhealing wounds.

## 1 Introduction

The management of acute and diabetic wounds constitutes a global health crisis, stalled by complex pathophysiology and exacerbated by the diabetes pandemic, which drives diabetic inflammation and healing impairment [1, 2, 3, 4]. This crisis imposes a staggering socioeconomic burden, with annual U.S. Medicare costs alone exceeding $96.8 billion [5, 6]. The critical insight, however, is that this cost is not dominated by advanced materials but by nursing labor, which constitutes 80%–85% of the total expenditure [7, 8].

This context reveals a fundamental dichotomy in wound care centered on the frequency of dressing changes. This single parameter embodies the tension between two competing therapeutic rationales: the principle of “Undisturbed Wound Healing” (UWH), which prioritizes the preservation of the wound's fragile microenvironment [9, 10], and the clinical necessity of aggressive intervention to disrupt the biofilm‐driven, inflammatory state of diabetic wounds [2, 4]. Consequently, the act of changing a dressing is inherently ambivalent—serving as both a critical debridement tool and a significant physiological stressor that can impede repair [11, 12].

Reconciling this dichotomy is a critical unmet need. Despite an era of “smart” materials [13, 14, 15, 16], their application remains governed by empirical tradition rather than evidence‐based science. This has resulted in a well‐documented knowledge gap; systematic reviews consistently report a lack of high‐quality evidence to inform optimal dressing selection or change frequency [17, 18]. This deficit is perpetuated by a research bias that prioritizes “materials over behavior,” creating a void in mechanistic preclinical data and leaving clinical practice reliant on standardized, “one‐size‐fits‐all” routines [10, 19]. Such protocols fail to account for the profound pathophysiological differences between acute and diabetic wounds, thereby creating the scientific imperative for the present study.

Our research is designed to resolve this dichotomy. We posit that the optimal dressing change frequency is not a static parameter but a dynamic variable dictated by the wound's evolving pathology. By leveraging parallel acute and diabetic murine models, we will, for the first time, systematically map the cellular and molecular consequences of varying dressing change frequencies. Our objective is twofold: to establish the first evidence‐based framework for clinical practice and to provide the foundational biological data needed to power the algorithms of next‐generation “smart dressings” [20]. Ultimately, this work aims to catalyze a paradigm shift, transforming wound care from an empirical art into a precision, intelligent science.

## 2 Methods

### 2.1 Experimental Animals

All animal experiments were approved by the Institutional Animal Care and Use Committee (IACUC) of Southern Medical University (Approval No: IACUC‐LAC‐20250415‐002) and conducted in accordance with the NIH Guide for the Care and Use of Laboratory Animals. Seven‐ to 10‐week‐old male C57BL/6J mice were housed in a full‐barrier, specific‐pathogen‐free (SPF) facility under a 12‐h light/dark cycle with ad libitum access to food and water. Mice were acclimated for 1 week prior to experiments.

### 2.2 Induction of Diabetes

Mice were fasted for 12 h, and baseline blood glucose was measured from the tail vein. Mice with glucose levels > 7 mmol/L were excluded. The remaining mice received an intraperitoneal (i.p.) injection of freshly prepared 1% streptozotocin (STZ, 55 mg/kg) in ice‐cold citrate buffer (pH 4.5). The control group received an equal volume of citrate buffer. One month postinjection, blood glucose was measured after a 7–8 h fast. Mice with blood glucose levels below 11.1 mmol/L were excluded to ensure a stable diabetic phenotype for the diabetic wound model.

### 2.3 Wound Healing Assessment

#### 2.3.1 Wound Healing in Normal Mice (Acute Model)

C57BL/6 mice (18–22 g) were randomly assigned to three groups (*n* = 5 per group): High‐frequency (daily), Intermediate‐frequency (every‐other‐day), and Low‐frequency (weekly) dressing changes. Following anesthesia, a 10‐mm full‐thickness circular dorsal wound was created. The wound was covered with a transparent occlusive dressing (Tegaderm). Wounds were photographed on days 0, 6, and 10. On days 3 and 10, a 3 × 3 cm square of skin tissue centered on the wound was harvested for analysis, and mice were euthanized. Half of each sample was fixed in 4% paraformaldehyde (PFA) for histology, while the other half was snap‐frozen for molecular analysis.

#### 2.3.2 Wound Healing in Diabetic Mice (Diabetic Model)

STZ‐induced diabetic mice were subjected to the same wounding procedure and assigned to the three frequency groups. Wounds were photographed on days 0, 6, 14, and 21. Tissue samples were collected on days 10 and 21 for histological analysis.

#### 2.3.3 Wound Closure Analysis

Digital photographs of wounds were taken with a 1 cm reference ring. Wound area was measured in a blinded manner using ImageJ software (v1.52a, NIH) and expressed as a percentage of the initial area.

### 2.4 Histological Analysis

Wound tissues were fixed in 4% PFA, paraffin‐embedded, and sectioned (5‐μm). Sections were stained with Hematoxylin and Eosin (H&E) to assess general tissue architecture and re‐epithelialization, and with Masson's trichrome to visualize collagen deposition (blue fibers).

### 2.5 mRNA Expression Analysis by qRT‐PCR

Total RNA was extracted from wound tissues using the E.Z.N.A. Total RNA Kit II. cDNA was synthesized using BlasTaq 2X PCR MasterMix, and qRT‐PCR was performed on a QuantStudio 7 Flex system. Gene expression was calculated using the 2^−^ΔΔCt method, with Gapdh as the reference gene. All experiments were performed in triplicate.

Inflammation & Macrophage Markers: Lbp (Lipopolysaccharide‐binding protein), Cd86 (M1 marker), Cd163, Cd206 (Mrc1) (M2 markers).

ECM Remodeling: Col1a1 (Collagen Type I Alpha 1).

### 2.6 Statistical Analyses

Data are presented as mean ± standard error of the mean (SEM). Statistical significance between three or more groups was determined using one‐way ANOVA followed by Tukey's post hoc test for multiple comparisons. A *p* < 0.05 was considered statistically significant. Analyses were performed using SPSS (v20.0).

## 3 Results

### 3.1 Intermediate‐Frequency Treatment Accelerates Healing in Acute Wounds

**FIGURE 1:**
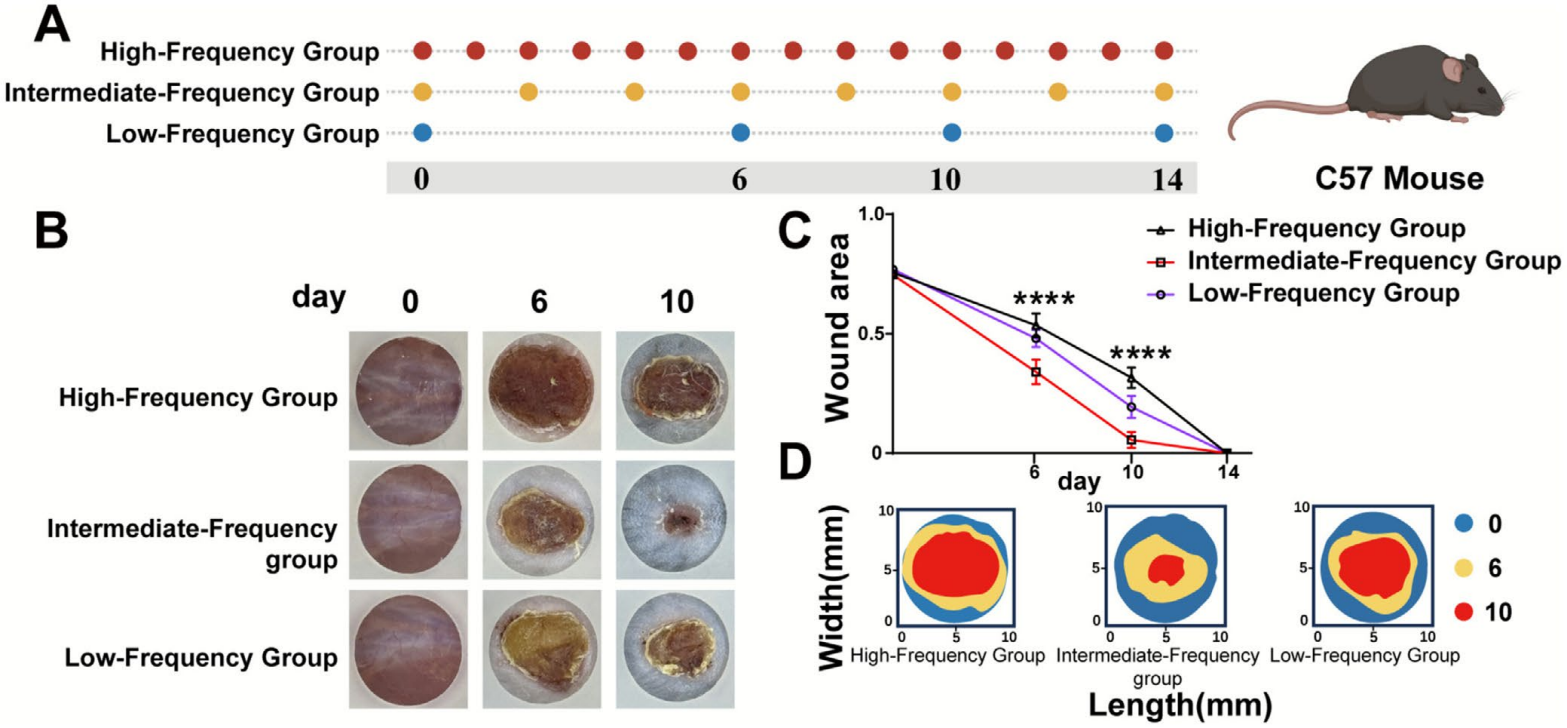
A‐D | Intermediate‐frequency treatment optimizes healing of acute murine skin wounds. (A) Experimental timeline. (B) Representative wound images on days 0, 6, and 10. (C) Quantification of wound closure. Data are mean ± SEM. ***p < 0.0001 vs. intermediate‐frequency group. (D) Wound contour plots showing perimeters on day 0 (blue), day 6 (yellow), and day 10 (red).

To define the optimal therapeutic frequency for acute wounds, we subjected C57 mice with full‐thickness skin defects to high‐, intermediate‐, or low‐frequency treatment regimens (Figure 1A). Macroscopic evaluation on days 6 and 10 revealed that the intermediate‐frequency group exhibited markedly accelerated closure and more robust epithelial coverage compared to both high‐ and low‐frequency groups (Figure 1B).

Quantitative analysis confirmed the superior kinetics of the intermediate‐frequency strategy (Figure 1C). By day 6, these wounds had contracted to ~35% of their original size, a significant improvement over the high‐ (~55%) and low‐frequency (~60%) groups (*p* < 0.0001). This advantage persisted at day 10, when the intermediate‐frequency group achieved near‐complete healing (< 10% area), while the other groups lagged significantly (*p* < 0.0001). Wound contour plots provided a clear visual representation of this rapid wound contraction dynamic (Figure 1D). These findings establish that an intermediate‐frequency (every‐other‐day) regimen is the optimal strategy for promoting rapid repair of acute skin wounds in this model.

### 3.2 High‐Frequency Treatment Is Essential for Healing in Diabetic Wounds

**FIGURE 2:**
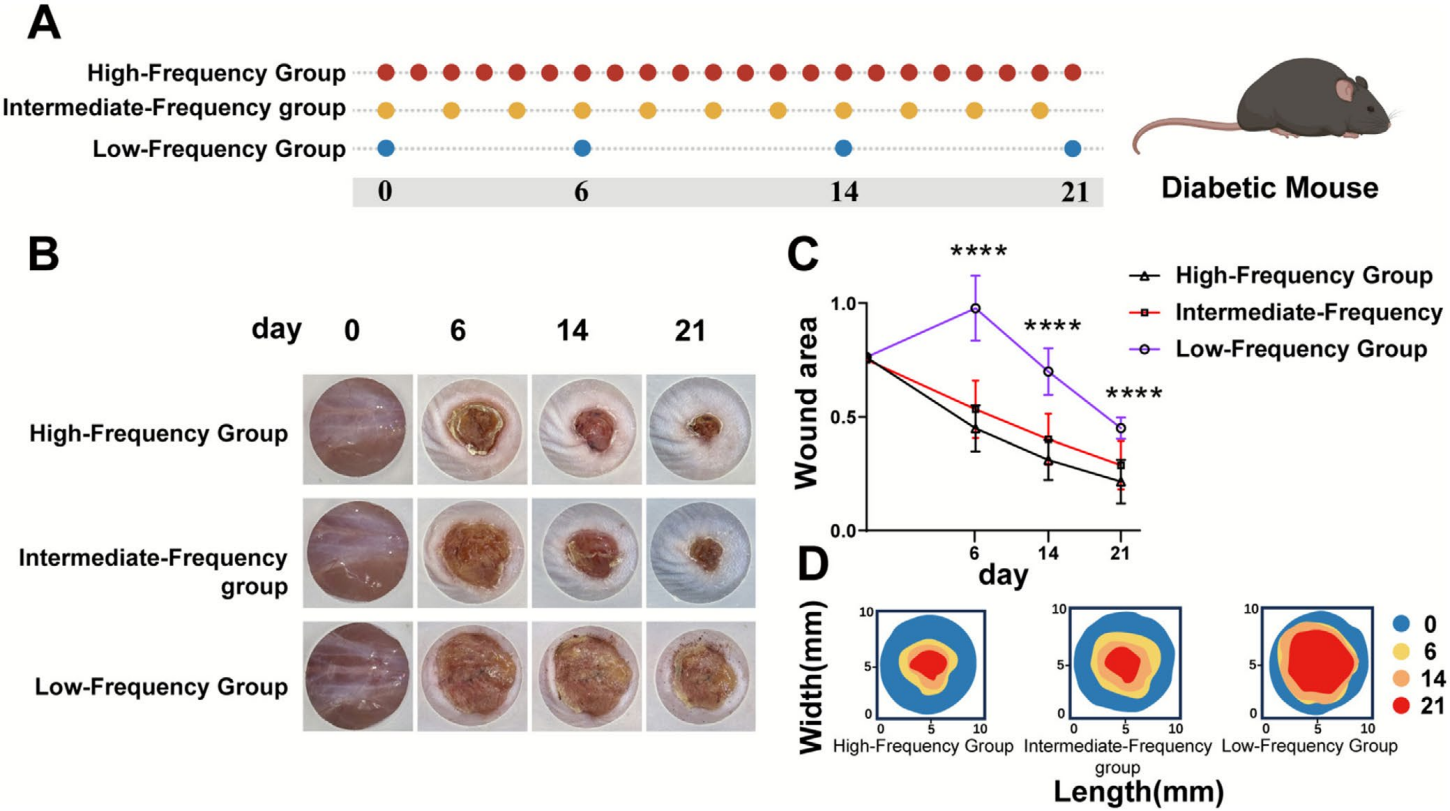
A‐D | High‐frequency treatment rescues impaired healing in a diabetic mouse model. (A) Experimental timeline. (B) Representative images of diabetic wounds on days 0, 6, 14, and 21. (C) Quantification of wound closure. Data are mean ± SEM. ***p < 0.0001, low‐frequency vs. high‐ and intermediate‐frequency groups. (D) Wound contour plots showing perimeters on day 0 (blue), day 6 (yellow), day 14 (orange), and day 21 (red).

In diabetic mice, the therapeutic requirement was starkly inverted. High‐frequency treatment drove a robust healing trajectory, achieving near‐complete re‐epithelialization by day 21 (Figure 2A,B). In dramatic contrast, the low‐frequency group suffered catastrophic healing failure. By day 6, these wounds had paradoxically expanded to ~100% of their original size—a profound deficit compared to the ~45% contraction in the high‐frequency group (*p* < 0.0001)—and remained critically stalled for the entire study (Figure 2C). These data unequivocally establish that for pathological wounds, a high‐frequency strategy is essential for repair, while infrequent intervention is not merely ineffective but actively harmful (Figure 2D).

### 3.3 Treatment Frequency Dictates Histological Regeneration and Collagen Deposition

**FIGURE 3:**
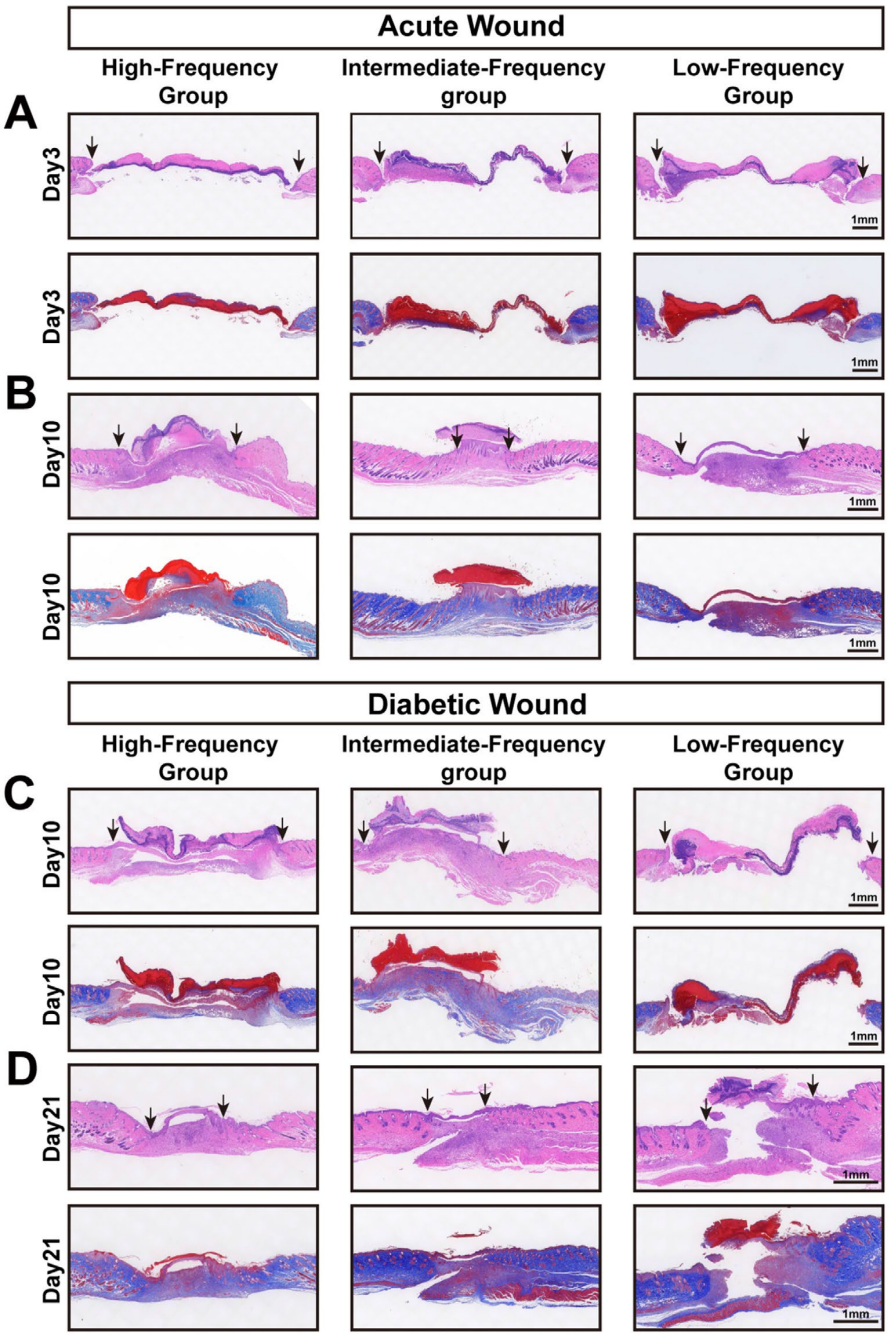
| Histological effects of treatment frequency on wound healing. A: Acute wound, Day 3; B: Acute wound, Day 10; C: Diabetic wound, Day 10; D: Diabetic wound, Day 21. Each panel includes H&E‐stained sections (top row) and Masson's trichrome‐stained sections (bottom row) for high‐, intermediate‐, and low‐frequency treatment groups. Arrows indicate wound edges, and scale bars represent 1 mm.

To investigate the tissue‐level impact of our interventions, we performed H&E and Masson's trichrome staining (Figure 3). The results provided a clear cellular basis for our macroscopic findings. In acute wounds, the intermediate‐frequency regimen resulted in complete re‐epithelialization and a dense, organized collagen matrix by day 10, significantly outperforming the high‐ and low‐frequency groups, which showed persistent epithelial gaps. In the diabetic model, however, high‐frequency treatment was required to achieve successful repair, yielding a mature, collagen‐rich matrix by day 21. The low‐frequency group failed to heal, displaying minimal re‐epithelialization and sparse collagen deposition. These findings confirm that intermediate‐frequency treatment is optimal for physiological tissue regeneration, while high‐frequency intervention is essential to drive matrix maturation in diabetically impaired wounds.

### 3.4 Frequency‐Dependent Modulation of Key Molecular Healing Pathways

To elucidate the molecular drivers of these frequency‐dependent outcomes, we performed qRT‐PCR analysis of key genes governing inflammation, macrophage polarization, and ECM remodeling (Table S1).

**FIGURE 4:**
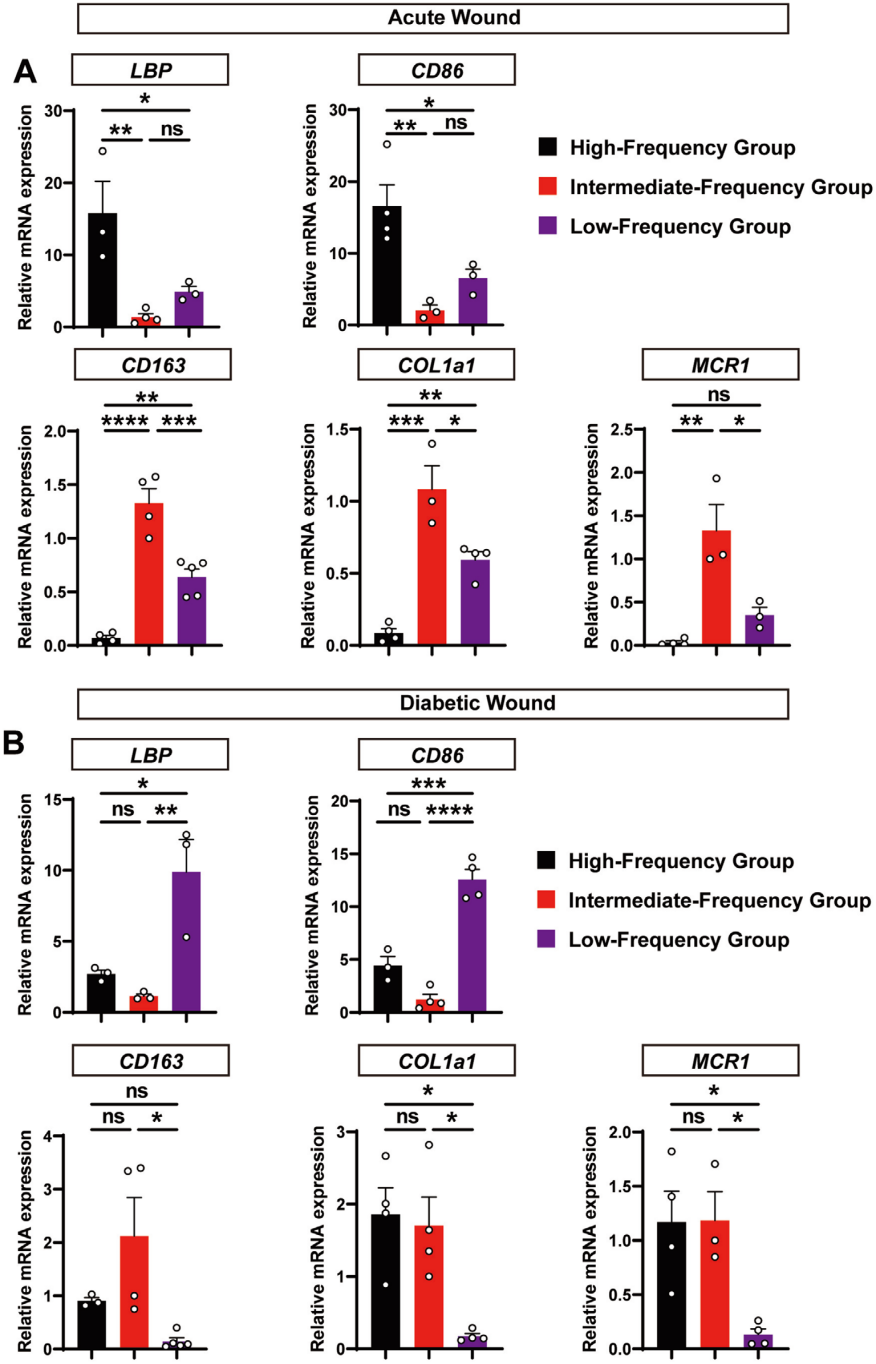
Gene expression analysis of key healing regulators in acute and diabetic wounds. Quantitative RT‐PCR was used to measure the relative mRNA expression of genes related to inflammation (LBP), macrophage polarization (Cd86, Cd163, Mcr1), and extracellular matrix (ECM) remodeling (Col1a1) in wound tissues. (A) Acute wound model (C57 mice, tissues harvested on day 10). (B) Diabetic wound model (tissues harvested on day 21). Data are presented as mean ± SEM (*n* ≥ 3 mice per group). Statistical significance was determined by one‑way ANOVA with Tukey’s post hoc test. *p < 0.05, **p < 0.01, ***p < 0.001, ****p < 0.0001; ns, not significant. Bar colors: black – high‑frequency group; red – intermediate‑frequency group; purple – low‑frequency group.

In acute wounds, the intermediate‐frequency regimen orchestrated an optimal healing cascade. It profoundly suppressed the expression of the pro‐inflammatory gene LBP and the M1 marker Cd86 to the lowest levels among all groups (Figure 4A). Concurrently, it unleashed a potent pro‐reparative program, driving the most significant upregulation of the M2 markers Cd163 and Mcr1. This ideal M1‐to‐M2 transition culminated in the highest expression of Col1a1, perfectly aligning with our histological findings of superior collagen deposition.

This therapeutic logic was inverted in the diabetic wound model, where the molecular signatures revealed a battle against pathological inflammation. The low‐frequency group succumbed to this challenge, exhibiting runaway expression of LBP and sustained high levels of Cd86, which locked the wound in a nonresolving inflammatory state (Figure 4B). This diabetic inflammation crippled matrix production, as evidenced by negligible Col1a1 expression. In stark contrast, the high‐frequency regimen successfully rescued the healing process. By suppressing the inflammatory markers and fostering a productive M2‐dominant environment, it drove a robust increase in Col1a1 expression, providing the molecular basis for the successful tissue regeneration observed in this group.

These data suggest that wound‐local LBP expression serves as a sensitive molecular barometer for inflammatory resolution. Its dynamic modulation is a key indicator of a therapy's ability to switch the microenvironment from a state of pathological defense to productive repair.

## 4 Discussion

This study establishes a fundamental principle of wound care: the optimal dressing change frequency is not a universal constant but a dynamic therapeutic variable. Our core contribution is the decoding of this frequency from a vague, experience‐based practice into a quantifiable, evidence‐based parameter. We demonstrate that its efficacy is governed by a sophisticated dual dynamic balance, which reconciles the seemingly contradictory needs of different wound types.

The first of these is the physicochemical balance between microbial control and tissue protection, which dictates the therapeutic imperative in diabetic/diabetic wounds. These wounds are often “locked” in a dysregulated inflammatory state driven by biofilms and compromised immunity [4, 21, 22, 23, 24]. Here, aggressive and frequent dressing changes function as an essential “macroscopic physical debridement” to break the bacteria‐inflammation vicious cycle and enable healing to commence [25, 26, 27].

The second is the psychophysiological balance between iatrogenic intervention and host stress, which explains why acute wounds demand protection [28]. In wounds with robust self‐healing capacity, daily changes proved detrimental [1]. We propose this is due to a “rhythmic physiological stress,” where frequent interventions continuously activate the stress‐inflammatory axis, trapping the wound in a nonresolving inflammatory state [11, 29]. The success of less frequent changes, therefore, lies not only in minimizing physical disturbance but in creating a physiologically “low‐stress” reparative environment, a concept supported by our findings of reduced pro‐inflammatory markers in the intermediate‐frequency group.

These findings have profound implications, fundamentally challenging entrenched clinical paradigms. They subvert the indiscriminate “ritualistic” daily dressing change routine, arming clinicians with a powerful evidence‐based rationale for personalizing care [10]. This approach offers a “win‐win” solution to the “multi‐stakeholder dilemma” [30], proposing a strategy that is biologically superior, more cost‐effective, and reduces patient and provider burden, aligning perfectly with value‐based healthcare principles [31].

Crucially, our work recalibrates the benchmark for evaluating all future wound care technologies. We demonstrate that a “zero‐cost” behavioral optimization can yield significant therapeutic gains. This demands a redefinition of the control group in clinical trials: any novel, expensive therapy must prove its value not against ambiguous “standard care,” but against standard care with optimized basic practices. This elevation of the gold standard is essential for correcting systemic biases that may overestimate new technology efficacy [32, 33] and for ensuring the scientific and ethical integrity of future research.

### 4.1 Limitations and Future Perspectives

In this study, we utilized a controlled animal model to decode dressing frequency as a quantifiable biological variable. However, we acknowledge that the optimal frequency is likely material‐dependent; therefore, our findings using Tegaderm may not directly apply to other dressing types. Furthermore, translation to complex clinical settings requires rigorous human trials. Nevertheless, these foundational principles illuminate three clear paths for optimization.

First, this work provides a direct rationale for optimizing clinical workflows. Simply modifying this basic “behavior” can significantly improve outcomes. This opens new avenues for exploring synergies between advanced dressing materials and change frequency. For example, can potent antimicrobial dressings like DACC [34] allow for safely extending change intervals, maximizing the benefits of UWH while conserving precious healthcare resources?

Second, our findings fundamentally recalibrate the benchmark for future clinical trials. We have shown that a “zero‐cost” intervention—optimizing frequency—yields significant therapeutic effects. This mandates that new, expensive therapies be compared against a control group receiving truly optimized basic care. This “elevation of the benchmark” is critical for accurate health economic assessments and for designing RCTs that genuinely guide clinical decisions.

Finally, this study provides the biological “soul” for next‐generation smart wound management, paving the way from “time‐based” to “needs‐based” intervention. The core of any “smart” system, be it a wearable sensor or a 3D‐printed dressing, is an intelligent decision algorithm [14, 35]. Our research provides the critical biological logic and “ground‐truth data” for such algorithms. We have defined the “if‐then” rules for switching between “intervene” and “protect” based on the wound's state: persistent inflammatory signals (e.g., high LBP) should trigger an alert to increase intervention, while pro‐reparative signals should trigger a protective, low‐disturbance mode. This framework is the indispensable theoretical cornerstone for realizing truly dynamic, individualized, and intelligent precision wound management [36, 37].

## Author Contributions

Ping He designed the study and wrote the original draft of the manuscript. Chencheng Mo and Hantao Li critically revised the manuscript for important intellectual content. Yiwen Han prepared the figures. Xiangdong Qi conceived and supervised the entire project, acquired funding, and provided final approval of the manuscript. All authors have read and agreed to the published version of the manuscript.

## Funding

This research was funded by the National Natural Science Foundation of China, 82472586 and the Guangdong Basic and Applied Basic Research Foundation, 2024A1515013286.

## Disclosure

Experimental Animals: All animal experiments were approved by the Institutional Animal Care and Use Committee (IACUC) of Southern Medical University (Approval No: IACUC‐LAC‐20250415‐002) and conducted in accordance with the NIH Guide for the Care and Use of Laboratory Animals. Seven‐ to 10‐week‐old male C57BL/6J mice were housed in a full‐barrier, specific‐pathogen‐free (SPF) facility under a 12‐h light/dark cycle with ad libitum access to food and water. Mice were acclimated for 1 week prior to experiments.

## Consent

The authors have nothing to report.

## Conflicts of Interest

The authors declare no conflicts of interest.

## Supporting information


**Table S1:** jocd70643‐sup‐0001‐Supplement.docx.

## Data Availability

The data that support the findings of this study are available from the corresponding author upon reasonable request.
