# Supplementary material for: Optimizing Wound Care: The Mechanistic Role of Dressing Change Frequency in Acute and Diabetic Wound Healing
Source: J Cosmet Dermatol. 2026 Jan 2;25(1):e70643. doi: 10.1111/jocd.70643 (PMC12759167; doi:10.1111/jocd.70643)
Supplement: Supplementary file 1 — Table S1: jocd70643‐sup‐0001‐Supplement.docx. [file JOCD-25-e70643-s001.docx]

**Supplementary Materials**

Primers are listed in Supplementary Table 1.Primer sequences were as follows:

| Gene | Species | Sequence (5'to3') |
| --- | --- | --- |
| Mrc1 | Mouse | Forward:5'- GTTCACCTGGAGTGATGGTTCTC-3'  Reverse:5'- AGGACATGCCAGGGTCACCTTT-3' |
| Lbp | Mouse | Forward:5'- TCCATCGGTGTCCGAGGCAAAT-3'  Reverse:5'- AGGTCCACTGAAATGGTGACACC-3' |
| Cd86 | Mouse | Forward:5'- ACGTATTGGAAGGAGATTACAGCT-3' Reverse:5'- TCTGTCAGCGTTACTATCCCGC-3' |
| Cd163 | Mouse | Forward:5'- GGCTAGACGAAGTCATCTGCAC-3'  Reverse:5'- CTTCGTTGGTCAGCCTCAGAGA-3' |
| GAPDH | Mouse | Forward:5'-CATCACTGCCACCCAGAAGACTG-3'  Reverse:5'-ATGCCAGTGAGCTTCCCGTTCAG-3' |
| Col1a1 | Mouse | Forward:5'-CCTCAGGGTATTGCTGGACAAC-3'  Reverse:5'-CAGAAGGACCTTGTTTGCCAGG-3' |
